# Neocortex expansion is linked to size variations in gene families with chemotaxis, cell–cell signalling and immune response functions in mammals

**DOI:** 10.1098/rsob.160132

**Published:** 2016-10-05

**Authors:** Atahualpa Castillo-Morales, Jimena Monzón-Sandoval, Alexandra A. de Sousa, Araxi O. Urrutia, Humberto Gutierrez

**Affiliations:** 1 Department of Biology and Biochemistry, University of Bath, Bath BA2 7AY, UK; 2 School of Life Sciences, University of Lincoln, Lincoln LN6 7TS, UK; 3 Milner Centre for Evolution, University of Bath, Bath BA2 7YA, UK; 4 Culture and Environment: Psychology, Bath Spa University, Bath BA2 9BN, UK

**Keywords:** neocortex, encephalization, brain size, gene family size

## Abstract

Increased brain size is thought to have played an important role in the evolution of mammals and is a highly variable trait across lineages. Variations in brain size are closely linked to corresponding variations in the size of the neocortex, a distinct mammalian evolutionary innovation. The genomic features that explain and/or accompany variations in the relative size of the neocortex remain unknown. By comparing the genomes of 28 mammalian species, we show that neocortical expansion relative to the rest of the brain is associated with variations in gene family size (GFS) of gene families that are significantly enriched in biological functions associated with chemotaxis, cell–cell signalling and immune response. Importantly, we find that previously reported GFS variations associated with increased brain size are largely accounted for by the stronger link between neocortex expansion and variations in the size of gene families. Moreover, genes within these families are more prominently expressed in the human neocortex during early compared with adult development. These results suggest that changes in GFS underlie morphological adaptations during brain evolution in mammalian lineages.

## 1. Introduction

Increased brain size in mammals when compared with other vertebrate taxa is thought to have played an important role in the expansion of this clade. Increased brain size during evolution has been previously related to increased behavioural complexity and the ability to cope with a changing environment [1,2]. However, the precise evolutionary drivers of brain size expansion in mammals and its relation to behavioural ability are still unclear and remain a topic of much interest and debate. This is complicated by the fact that different mammalian clades have differences in the degree of size-related changes in brain tissue [3]. Generally, large brains differ from small brains in having larger neuronal soma sizes [4], increased numbers of non-neuronal cells, in particular glia [5,6], and lower overall neuron density [7]. Large brains, however, are associated with a high metabolic cost [8–11] as well as higher demands of parental investment and delayed sexual maturation [12–16].

Brain size is a highly variable trait among mammalian and non-mammalian species with marked differences observed even between relatively close species [17–21]. Because brain size is closely associated with variations in body mass across species [22], comparative studies of brain size frequently use a corrected measure of brain size, known as encephalization index (Ei), which provides a measure of how much brain size is above (or below) what is expected for a given body size. While Ei is commonly regarded as an index that aligns more closely with behavioural capacity [1,23,24], many studies have also related behavioural complexity directly to the actual size of specific brain regions as well as to relative brain size as a whole [25–29]. Changes in relative brain size (or Ei) on the other hand, are not necessarily the result of a proportional expansion of all brain structures. In many mammalian lineages, most variations in encephalization index are closely linked to changes in the size of the neocortex [30–32], a distinctive structure of the mammalian brain and one of the most salient evolutionary innovations of the mammalian lineage [33–36].

The characteristic increase in the size of the neocortex relative to the rest of the brain has long been considered one of the primary targets of selection during mammalian brain evolution [37–39]. Increases in the absolute size of the neocortex are related to an increase in the number of functionally distinct neocortical areas [40–42], potentially allowing more complex information processing and the emergence of new behaviours [43]. In comparative studies in primates, for instance, relative size of the neocortex has been correlated with social group size [12,44,45] (but see [46]), and it has been speculated that the number of neocortical neurons may be a limiting factor in determining the number of social relationships mammals can effectively establish and manage [44]. More neocortical areas may be found in larger brains due to the lower marginal cost of devoting additional neural tissue to increasingly specialized functions [47], and an increasing number of neocortical areas may facilitate a more elaborate processing of sensory and motor information [48,49].

In the hominid lineage, the expansion of the neocortex is thought to have played a key role in the evolution of modern humans [50], including specialized areas involved in processing and production of language [51,52] as well as areas involved in identification of faces [53,54] and locations [55,56]. The neocortex in humans is widely regarded as the primary seat for the so-called higher cognitive functions, including self-awareness, consciousness, abstract reasoning and planning [57–63]. Development of the neocortex extends well into adolescence in humans and, although the structure of the layers in the neocortex is established during early prenatal development [64], the neocortex keeps growing in childhood and adolescence, reaching a peak in thickness on average at around 13 years of age, while myelination of some cortical regions can still continue after 20 years of age [65].

Despite the importance of the neocortex, the genomic features underlying its expansion during mammalian evolution remain poorly understood [30,66,67]. So far, there have been few efforts to identify features reflecting the genomic impact of brain evolution. Dorus and co-workers [68] reported accelerated sequence evolution of genes functioning in the nervous system during human origins, but this pattern was contested by later studies [69,70]. A genome-wide analysis of amino acid composition across 37 fully sequenced mammalian genomes showed that encephalization is significantly correlated with overall protein amino acid composition, although the causes of this pattern remain unclear [71].

Changes in gene family size (GFS) can reflect changes in the relative relevance of specific functions in an organism. Gene duplication events have been proposed to play a major role in the origin of novel gene functions and expression patterns [72,73]. Marked differences in GFS have been identified in *Drosophila* and vertebrates, with families experiencing the largest changes being enriched in distinct biological functions [74–76]. Among mammals, marked differences in the number of olfactory receptors are likely to reflect variations in the reliance of different lineages on their sense of smell [77–80]. A recent study found that encephalization in mammalian lineages is associated with significant variations in GFS, with a significant enrichment of genes associated with immune system response, chemotaxis and cell–cell signalling functions among the most positively associated gene families [81]. Here, we investigate if variations in the relative size of the neocortex or neocortex to brain size ratio (Nr) are associated with changes in GFS in mammalian lineages, and whether the extent to which any changes in GFS associated with Nr could explain previously reported associations between GFS variations and encephalization. We further explored whether any associated correlations between Nr and GFS are functionally reflected by the specific patterns of expression of Nr-associated families in the developing neocortex in humans.

## 2. Material and methods

### 2.1. Gene family annotations

Annotated gene families encompassing 28 fully sequenced mammalian genomes were obtained from Ensembl release 76 [82] (http://www.ensembl.org. Ensembl release 76). In the context of this annotation, Ensembl families are defined by clustering all Ensembl proteins along with metazoan sequences from UniProtKB. Any given gene family constitutes a group of related genes that includes both paralogues within the same species and orthologues and paralogues from other species. Any given gene can only be assigned to a single gene family. GFS in a given family for a given species was calculated as the total number of member genes contained in that gene family, for that particular species. In this study, we included all gene families with members present in at least six of the 28 mammalian species (*n* = 11 943). We excluded from this study any gene family with no variance in GFS across species.

### 2.2. Phenotype data

Body mass-corrected values of brain mass, known as encephalization index (Ei), were computed as
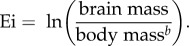
The slope (*b*) was estimated as 0.64 [83] based on a log–log least-squares linear regression of brain mass against body mass data from 493 mammalian species (table 1). Neocortex volumes were compiled from available literature (table 1), and include the grey and white matter of the cerebral cortex. Grey matter from palaeocortical structures (entorrhinal cortex, schizocortex, hippocampus and amygdala) were excluded. Nr was defined as
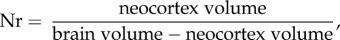
after Dunbar [44]. Maximum lifespan (MLSP) for each species was obtained from the animal ageing and longevity database, AnAge [100]. Brain region volumes and corresponding sources as well as encephalization indexes and MLSP for all included species are shown in table 1.

**Table 1.** Phenotypic traits for the 28 mammalian species analysed.

species name	common name	non-neocortex brain volume (cm^3^)	neocortex volume (cm^3^)	ref.	Nr	Ei	MLSP
*Ailuropoda melanoleuca*	giant panda	211.80935	136.43571	[84]	1.81	−2.014	36.8
*Callithrix jacchus*	marmoset	7.241	4.371	[85]	1.52	−1.627	16.5
*Canis familiaris*	dog (poodle)	458.273	177.753	[86]	0.63	−1.699	24
*Cavia porcellus*	guinea pig	4.671815	1.5798	[87]	0.51	−2.948	12
*Echinops telfairi*	lesser hedgehog tenrec	0.566	0.0515	[85]	0.1	−3.274	19
*Erinaceus europaeus*	hedgehog	3.05	0.522	[85]	0.21	−2.863	11.7
*Gorilla gorilla*	gorilla	470.359	341.444	[85]	2.65	−1.415	55.4
*Homo sapiens*	human	1251.847	1006.525	[85]	4.1	0.152	122.5
*Loxodonta africana*	elephant	3886.7	2460.1	[88]	1.72	−1.082	65
*Macaca mulatta*	macaque	87.896	63.482	[85]	2.6	−1.192	40
*Macropus eugenii*	wallaby	11.6637	4.3987	[89]	0.61	−2.207	15.1
*Microcebus murinus*	mouse lemur	1.68	0.74	[85]	0.79	−1.985	18.2
*Mus musculus*	mouse (C57BL/6J)	0.48	0.12	[90]	0.32	−2.832	4
*Mustela putorius furo*	European polecat	8.8996	4.147	[91]	0.87	−2.548	11.1
*Ornithorhynchus anatinus*	platypus	8.57145	4.09928	[92]	0.92	−2.219	22.6
*Ovis aries*	sheep	100.332	53.793	[93]	1.16	−1.961	22.8
*Pan troglodytes*	chimpanzee	382.103	291.592	[85]	3.22	−0.948	59.4
*Papio anubis*	olive baboon	190.957	140.142	[85]	2.76	−1.178	37.5
*Pongo abelii*	orangutan	304.2	219.8	[94]	2.6	−0.892	59
*Procavia capensis*	hyrax	12.68	5.54	[95]	0.78	−2.255	14.8
*Pteropus vampyrus*	megabat	8.89	3.61	[96]	0.68	−2.204	20.9
*Rattus norvegicus*	rat	1.69	0.58	[95]	0.52	−2.861	5
*Sarcophilus harrisii*	Tasmanian devil	15.1517	3.7334	[89]	0.33	−2.792	13
*Sorex araneus*	shrew	0.188	0.0264	[85]	0.16	−2.832	3.2
*Sus scrofa*	pig	106.660	54.3913	[97]	1.04	−2.468	27
*Tarsius syrichta*	tarsier	3.393	1.768	[85]	1.09	−1.795	16
*Tursiops truncatus*	dolphin	1376.976	1088.615	[98]	3.78	−0.321	51.6
*Vicugna pacos*	alpaca	181.467	101.81	[99]	1.28	−1.688	25.8

### 2.3. Correlation coefficients of gene family size and different phenotypes

Pearson's correlations between GFS values and the three phenotypes, Ei, Nr or MLSP, for all 11 943 gene families included in the study, were calculated using R-based statistical functions. To determine the statistical significance of any potential shift in the distribution of Pearson's correlation coefficients when compared to random expectation, 10 000 Monte Carlo simulations of the expected distribution based on random permutations of GFS values across species were conducted and contrasted with the observed distribution of correlation coefficients using a *Z*-score test.

### 2.4. Confounding variables and phylogenetically controlled correlations

**Figure 1.**
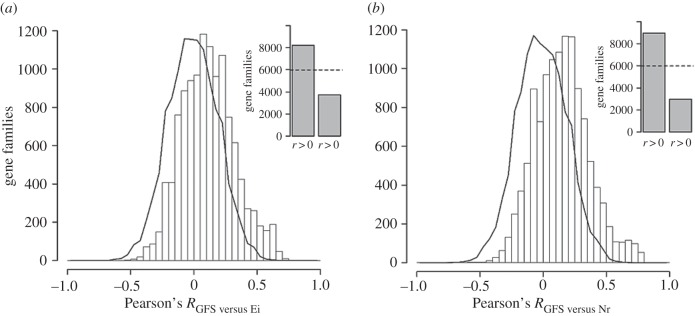
Enrichment of gene family size variations (GFS) in line with increased encephalization index (Ei) and neocortex to brain size ratio (Nr) in mammals. (*a*) Histogram showing the distribution of correlation coefficients for GFS and Ei in 11 943 gene families encompassing 28 mammalian genomes. (*b*) Histogram showing the distribution of correlation coefficients for GFS and Nr in 11 943 gene families encompassing 28 mammalian genomes. In each figure, an estimation of the expected distribution derived from 10 000 Monte Carlo simulations is represented by the solid line. Inset: distribution of positive and negative correlations relative to the expected distribution (dashed line).

In order to remove the effect of Ei and MLSP on Nr, we calculated residuals for the multivariate regression of Nr ∼ Ei + MLSP (with Nr as the response variable and Ei and MLSP as independent covariates). For consistency, we used the exact same approach to obtain similar corrected estimates for all GFS values after correcting for any potential effects of Ei and MLSP. This was done by extracting the residuals for the multivariate regression GFS ∼ Ei + MLSP for each individual gene family. The resulting sets of residuals where then used to obtain phylogenetic independent contrasts (PIC) to further account for any effect of phylogenetic relationships on these variables [101]. The resulting independent contrasts were finally used to assess the final corrected association between Nr and GFS by simply using standard Pearson's correlations forced through the origin. The same analysis was carried but using Nr and MLSP instead as independent covariates to generate residuals for all Ei and GFS values, from the multivariate regressions Ei ∼ Nr + MLSP and GFS ∼ Nr + MLSP respectively, followed by extraction of the corresponding PIC to assess the unbiased association between Ei and GFS (figure 1). PIC analysis was computed using the ape package in R [102]. Ultrametric phylogeny of the 28 analysed mammalian species was obtained from TimeTree database [103] (http://www.timetree.org/. TimeTree2).

### 2.5. Gene Ontology term enrichment

Gene Ontology (GO) annotations per species for biological process domains were obtained from Ensembl's Biomart release 76 [82]. A GO term was associated with a family whenever that term was linked to any of its members in any species. To minimize the effect of very small functional categories, only terms linked to at least 200 gene families were examined (*n* = 116). GO terms with less than 200 gene families were assigned to a ‘small biological process GO terms’ category while gene families not annotated to any GO term in any species were grouped into a ‘not annotated’ category. Enrichment analysis of these GO terms was carried out as described in Castillo-Morales *et al*. [81]. Briefly, over-representation of genes associated with specific GO terms was assessed by counting the number of gene families assigned to each GO term within the analysed set of gene families. Statistical significance was numerically assessed by obtaining the expected number of families per GO in 1000 equally sized random samples derived from the overall population of gene families. Because genes vary in the number of GO terms associated with them, we adjusted for differences in the density of GO annotations between the test and background samples, by dividing the family counts per GO from each sample by the sample's average number of GO annotations per family.

### 2.6. Gene expression before and after full cortical maturation

RNAseq RPKM normalized expression data summarized to genes were obtained from the NIMH Transcriptional Atlas of Human Brain Development database [104] (http://brainspan.org. BrainSpan Atlas of the Developing Human Brain) for a total of 143 post-mortem human brain samples corresponding to 11 cortical regions across 13 different ages. The cortical regions include primary auditory cortex (core) (A1C), dorsolateral prefrontal cortex (DFC), posteroinferior (ventral) parietal cortex (IPC), inferolateral temporal cortex (area TEv, area 20) (ITC), primary motor cortex (area M1, area 4) (M1C), anterior (rostral) cingulate (medial prefrontal) cortex (MFC), orbitofrontal cortex (OFC), primary somatosensory cortex (area S1, areas 3,1,2) (S1C), posterior (caudal) superior temporal cortex (area TAc) (STC), primary visual cortex (striate cortex, area V1/17) (V1C) and ventrolateral prefrontal cortex (VFC). The samples covered developmental stages 16, 24, 37 post-conception weeks, four months after birth and 1, 3, 8, 13, 19, 21, 30, 36 and 37 years old. Gene expression data were further normalized against the total expression per sample, and divided into two developmental groups, corresponding to the periods before and after full maturation of cortical thickness, which occurs at about 13 years of age in humans [65]. For each gene, expression levels were averaged across stages and structures of the same developmental window and comparisons between developmental windows were carried out by means of paired Wilcoxon tests.

## 3. Results

In order to assess the association between gene family size, GFS, and neocortex expansion, Nr, values were compiled from the literature for 28 mammalian species with fully sequenced genomes (table 1). GFS was calculated for a total of 11 943 non-overlapping families. Pearson's correlation coefficients between GFS and Nr were then calculated for each gene family. We found a significant over-representation of gene families with positive associations between GFS and Nr (figure 1) (*χ*^2^ = 2973.263083, *p* < 1 × 10^−20^). A Monte Carlo simulation showed that the observed shift in the distribution towards positive values is statistically significant when compared with random expectation (*Z*-score for observed mean *R* = 2.225819868, *p* = 0.013).

**Figure 2.**
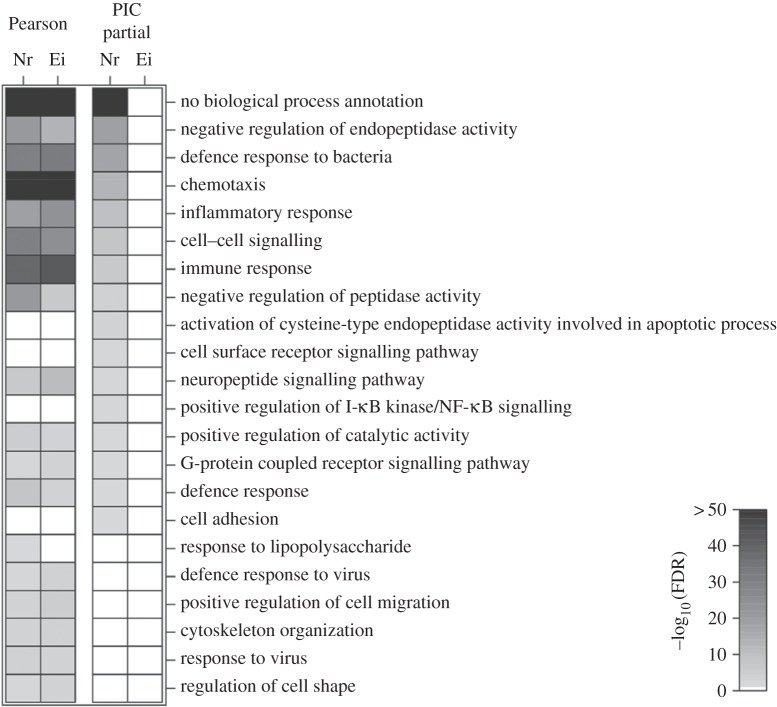
Gene ontology enrichment analysis of families with gene family size (GFS) variations in line with encephalization index (Ei) and neocortex to brain size ratio (Nr). Heatmap of the significance of the over-representation of GO terms (expressed as Benjamini–Hochberg (BH)-corrected *p*-value) among gene families most significantly associated with Ei and Nr. First two columns correspond to gene families with the most significant association between GFS and Ei or Nr, respectively (*r*_Nr GFS_ > 0, FDR < 0.05, *n* = 440 and *r*_Ei GFS_ > 0, FDR < 0.05, *n* = 321). Third and fourth columns represent GO terms enriched among gene families whose GFS variations display the most significant association with one of the brain phenotypes after accounting for the shared variance with the other neural phenotype, as well as the phylogenetic relationship of the analysed species using independent contrast analysis (*r*_PIC(Nr∼Ei+MLSP),PIC(GFS∼Ei+MLSP)_ > 0, FDR < 0.05, *n* = 272 and *r*_PIC(Ei∼Nr+MLSP),PIC(GFS∼Nr+MLSP)_ > 0, FDR < 0.05, *n* = 0 respectively). Only GO terms significantly enriched after BH multiple testing correction are shown in the figures.

In order to assess whether the observed bias towards strong correlations between GFS and Nr preferentially involves gene families associated with specific biological functions (as opposed to random sets of gene families), we assessed the statistical over-representation of functional annotations (annotated GO terms per gene family, see Material and methods) for the 440 gene families found to be significantly associated with Nr (*r*_Nr, GFS_ > 0 and FDR < 0.05). A total of 18 GO functional categories were found to be significantly enriched (FDR < 0.05) among Nr-associated gene families including immune response, negative regulation of endopeptidase activity, chemotaxis, cell–cell signalling, neuropeptide signalling pathway and G-coupled receptor signalling pathway (figure 2). Notably, genes with no functional annotations showed the highest over-representation.

As Nr is known to be highly correlated to relative brain size, the observed association between Nr and changes in GFS could be explained in principle by a previously reported association between GFS and relative brain size [81]. Indeed, after calculating correlation coefficients between GFS and Ei (a commonly used index of brain size relative to body mass) for each gene family in the same set of 28 species used in this study (figure 1), we also found a significant shift in the distribution favouring positive associations. This finding is consistent with a previously reported study using a larger set of 39 species [81]. The shift in the distribution of correlation coefficient values, however, was found to be stronger for Nr compared with Ei with the significance of the deviation for the latter being lower, (*Z*_Ei_ = 1.70943, *p* = 0.044, figure 1). Functional annotation enrichment analysis revealed a total of 17 GO term categories enriched among the set of gene families found to be significantly associated with Ei (*r*_Ei, GFS_ > 0 and FDR < 0.05), with a strong overlap with the 18 GO functional categories found overrepresented among Nr-associated gene families (Jaccard index = 0.67) (figure 2).

To assess whether variations in GFS associated with Nr are secondary to the relationships between Ei and GFS, we obtained Ei-corrected residuals for Nr and GFS. In addition, due to a known relationship between encephalization and MLSP in mammals we also corrected for the potential effect of this trait [83,105]. Finally, in order to remove any phylogenetic signal from the correlations between our traits of interest and GFS arising from interrelatedness among species, we used the above Nr and GFS residuals to conduct a PIC analysis (see Material and methods). This phylogenetically corrected analysis of GFS and Nr residuals revealed a total of 272 families significantly associated with Nr after correction for multiple testing (phylogenetically controlled *r*'s > 0, FDR < 0.05; electronic supplementary material, table S1). By contrast, phylogenetically controlled correlations between equivalent GFS and Ei residuals (correcting for the effect of Nr and MLSP, see Material and methods) resulted in no gene families with a significant association after correcting for multiple testing. Nr-associated gene families after this correction against confounding variables were found to be enriched in GO terms including immune response, negative regulation of endopeptidase activity, chemotaxis, cell–cell signalling and neuropeptide signalling pathway (figure 2).

If the association between GFS and Nr responds to the functional demands imposed by the development of a large neocortex, we should expect genes associated with families displaying a high correlation with Nr to also display a pronounced level of activity prior to full cortical maturation (when full cortical thickness is reached), compared with later stages. To this end, we used available gene expression data derived from human neocortex obtained from the BrainSpan Atlas of the Developing Human Brain [104] (see Material and methods). We found that gene members of this set of families showed higher expression levels during human development prior to the neocortex reaching maximum thickness (which in humans occurs around the age of 13 years) compared with later stages, reflecting a transcriptional signature of the potential involvement of some of these genes in the development of the neocortex (*p* = 0.00013).

## 4. Discussion

The expansion of the neocortex observed in several mammalian lineages is considered to be linked to a proliferation of new cortical areas driving increased cognitive capabilities [40,106]. The genomic drivers shaping the evolution of the brain and its morphology remain, however, poorly understood. By comparing the genomes of 28 mammalian species, here we have assessed the potential association between changes in GFS and the expansion of the neocortex. We show that neocortical expansion is indeed strongly and specifically associated with variations in GFS in mammals. Furthermore, variations in relative neocortical size account for a high proportion of the previously reported links between GFS and changes in encephalization across mammalian species [81]. This suggests that changes in GFS in line with relative brain size in mammals are actually secondary to the underlying correlation between neocortex size and encephalization. Analysis of available human neocortex gene expression data revealed that genes in families strongly and specifically associated with neocortex size variations also show significantly higher levels of expression at stages of development before (but not after) maximal cortical development is reached in humans, thereby supporting a functional role for these gene families in the ontological development of a large neocortex. Among the 272 gene families whose size was found robustly correlated with relative neocortex size, even after correcting for encephalization, MLSP and phylogenetic relationships, 16 distinct biological functions (GO terms) were found to be significantly overepresented. Among these, cell–cell signalling and chemotaxis are known to play critical roles in the development and maintenance of the nervous system. Example of Nr-associated gene families annotated to these functions are the tyrosine kinase precursor family (ENSFM00730001521921), encoding receptor protein-tyrosine kinases and widely known to promote cell survival, proliferation, adhesion and migration in the central nervous system [107–109]. The leukotriene B4 receptor 2 family (ENSFM00680001303697) includes leukotriene B4, a proinflammatory signalling molecule which has been shown to mediate regulation of neural stem cell proliferation and differentiation [110].

Several immune-related biological functions (inflammatory response, defence response to bacteria, immune response, defence response and positive regulation of I-κB kinase/NF-κB signalling) were also enriched among Nr-associated gene families. Along these lines in recent years, numerous immune-related signalling and regulatory components have also been shown to play key physiological roles in the developing and adult nervous system (for a review see [111]). This involvement of individual immune-related signalling components in neural functions has been shown to be part of a wider genetic network of immune-related molecules acting as an intrinsic component of the neural-specific regulatory machinery that ultimately shapes the normal development of the nervous system [112]. Thus, for instance, members of the tumour necrosis factor (TNF) receptor superfamily (ENSFM00500000273041, a gene family found to be highly associated with neocortex expansion here), are themselves part of the extensively studied canonical pathway of activation of the transcription factor NF-κB during early development of the nervous system [111].

Interestingly, gene families with no reported functional annotations for any of its members in any species showed the highest enrichment among the gene families with the highest positive associations with relative neocortex size. Among these families, we found the neuroblastoma breakpoint gene family (ENSFM00250000000879), whose members contain DUF1220 domains. DUF1220 domains have been previously linked to brain and cortical expansion in primate species, particularly in the human lineage [113,114]. Polymorphic deletions and duplications of DUF1220 domains have been associated with brain size variations in normal individuals from different human populations as well in pathological cases including microcephaly and macrocephaly [115,116]. Moreover, it has been proposed that proteins containing this domain have an important role during cortical neurogenesis, as they promote proliferation in neural stem cells [113], and during normal development they are expressed in the sub-ventricular zone precisely during the period of cortical neurogenesis [114].

Of particular importance to build a larger neocortex is the control of successive rounds of proliferation during early development, where the interplay between symmetric and asymmetric cell division is thought to be critical in shaping the particular morphology of the neocortex [117]. Consistent with this, one gene family with significant GFS changes in line with increased relative neocortex size is the ENSFM00250000003440 gene family of epithelial cell adhesion molecules, which in turn include known human developmental regulators such as EPCAM and TACSTD2. EPCAM has been shown to be involved in cell proliferation, differentiation and migration in diverse cell types [118,119] and could thus play an important role in neocortex development. A more numerous gene family found was the speedy gene family (ENSFM00740001589497), which encodes proteins able to bind CDKs but having no similarity with cyclins, and some of its members are known to play a role in the regulation of cell cycle [120,121]. While a great variation in gene numbers across species has been documented in this family [122], here we report the first evidence of a strong association between these variations in this family and relative neocortex size in mammals.

## 5. Conclusion

In summary, we have identified a set of gene families whose sizes are positively associated with an expanded neocortex, providing new insights into neocortex evolution. Moreover, as aberrant development and degeneration of cortical neurons has been linked with a variety of mental health pathologies and dementias [123,124], identifying genomic signatures associated with the evolution of larger brain size and neocortex expansion will critically contribute to our understanding of the molecular pathways involved in the development and maintenance of cortical areas in highly encephalized mammals including humans. As these pathways may not be present or developed to the same extent in less encephalized mammalian species, our finding could help to fill existing gaps in current knowledge gained from widely used rodent models.

## Supplementary Material

Supplementary Table 1

## Data accessibility

The electronic supplementary material, table S1 shows gene families significantly associated with Nr after correcting for the effect of Ei and MLSP and phylogenetic relatedness.
